# Acute kidney injury after cardiac arrest: the role of coronary angiography and temperature management

**DOI:** 10.1186/s13054-019-2476-8

**Published:** 2019-05-30

**Authors:** Angélique M. E. Spoelstra-de Man, Heleen M. Oudemans-van Straaten

**Affiliations:** Department of Intensive Care Medicine, Research VUmc Intensive Care (REVIVE), Amsterdam Cardiovascular Sciences (ACS), Amsterdam Infection and Immunity Institute (AI&II), Amsterdam University Medical Centers, Location VUmc, De Boelelaan 1117, 1081 HV Amsterdam, The Netherlands

**Keywords:** Cardiac arrest, Acute kidney injury, Coronary angiography, Percutaneous coronary intervention, Hypothermia

Cardiac arrest (CA) leads to acute kidney injury (AKI) in 12–81% of the patients [1]. Underlying disease, decreased renal perfusion due to shock and use of nephrotoxic medication can all impair renal function. AKI after CA is associated with higher mortality, poor neurological outcome, increased dialysis requirements and prolonged hospital stay [2, 3]. After CA, coronary angiography with percutaneous coronary intervention (CAG-PCI)) and targeted temperature management (TTM) are applied to improve myocardial function, survival, and neurological outcome. Both interventions have miscellaneous cardiovascular and renal effects and it is unclear whether their net effect on renal function is beneficial or harmful (Fig. 1).

**Fig. 1:**
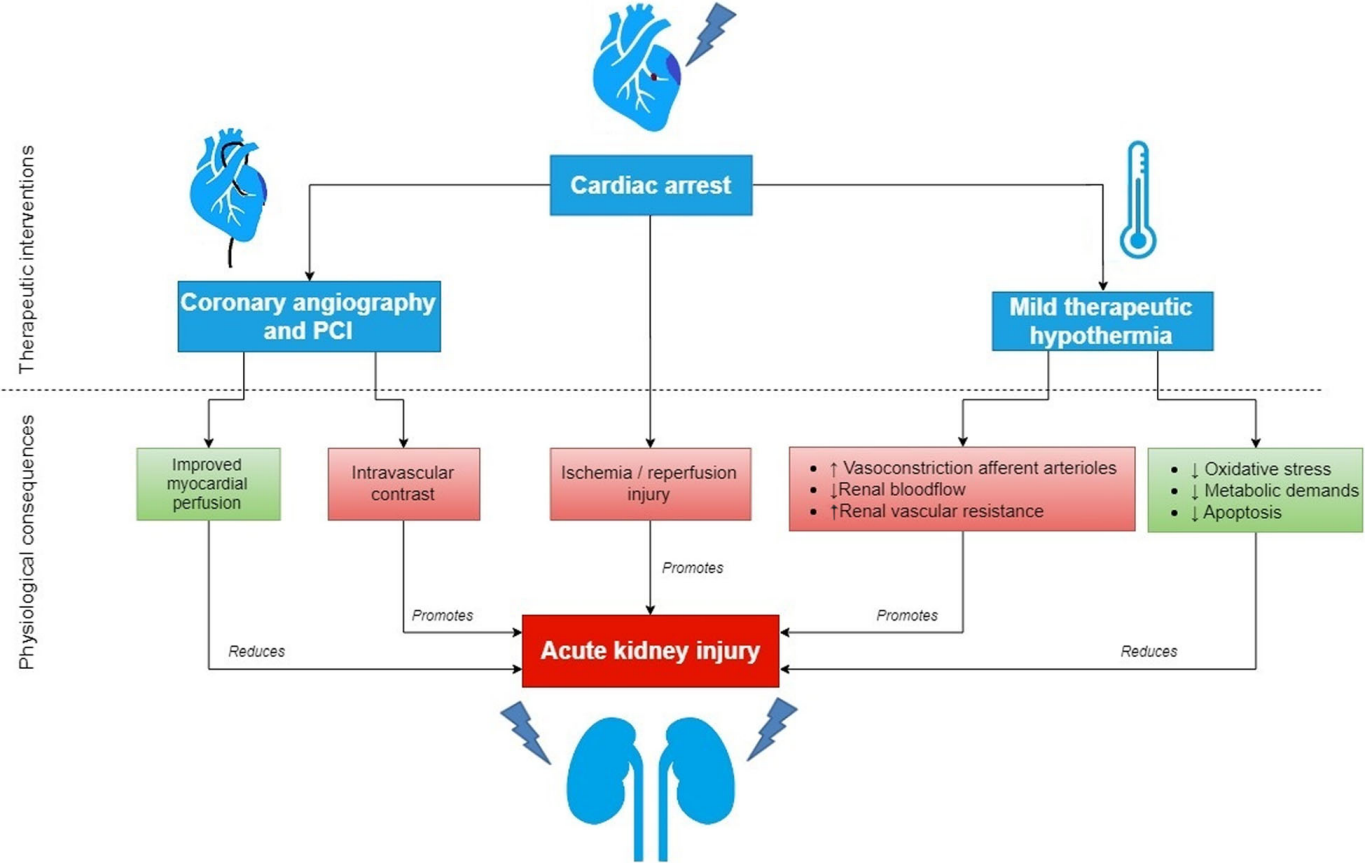
The physiological consequences of therapeutic interventions post-cardiac arrest on acute kidney injury

## Coronary angiography with percutaneous coronary intervention

The aim of CAG-PCI after CA is to detect and immediately treat the acute culprit coronary lesions. Potential subsequent improved myocardial perfusion can decrease infarct size, stabilize hemodynamics and reduce the incidence of recurrent cardiac arrest, which can all contribute to better neurological outcome [4]. Faster recovery of cardiac function may also improve renal perfusion. However, the iodinated contrast administered during CAG-PCI may cause tubular damage due to renal vasoconstriction with potential hypoxia and direct cytotoxic effects. Therefore, clinicians are concerned that the potential harm of contrast exposure may prevail over the possible benefit of increased renal perfusion.

Current guidelines recommend urgent CAG-PCI for all CA patients presenting with ST-elevation myocardial infarction (STEMI), but there is an ongoing debate whether this procedure in CA patients with non-STEMI should be performed immediately or postponed until the patient has stabilized. Current guidelines recommend immediate CAG-PCI only in selected CA-patients with non-STEMI, i.e., in those with hemodynamic or electrical instability, evidence of ongoing ischemia, or perceived high likelihood of cardiac etiology undergo [5]. Only patients with acute unstable lesions will benefit from immediate CAG-PCI. The reported incidences of acute instable lesions in CA patient with non-STEMI vary from 17 up to 58%. A meta-analysis in 2017 showed that early CAG-PCI in patients with non-STEMI decreased mortality and improved neurological outcome [6]. However, this meta-analysis mostly included retrospective observational studies and just 1 small RCT (36 patients). In the recent COACT trial immediate CAG-PCI did not improve survival and neurological outcome in 552 post-CA patients with non-STEMI compared to delayed CAG-PCI [7]. However, whereas 64.5% of the patients had coronary artery disease, only 15% had acute instable lesions and 5.0% acute thrombotic occlusions. So, the number of patients who could potentially benefit from immediate CAG-PCI was low. Future trials with more specific inclusion criteria may identify subgroups of CA-patients with non-STEMI with a higher probability of acute instable lesions who could potentially benefit from urgent CAG-PCI.

Recent studies show that early CAG-PCI does not increase the risk of AKI. In the COACT trial, the incidence of AKI and need for renal-replacement therapy did not differ between early vs. late CAG-PCI [7]. In the post hoc analysis of the targeted temperature management (TTM) trial, early CAG-PCI was even associated with less AKI [1]. Forty-two percent of the CA patients undergoing early CAG-PCI developed AKI, compared to 50% of the patients with late/no CA-PCI (*p* = 0.014) with worse severity of AKI in the late/no CAG-PCI group (*p* = 0.007). However, groups were not randomized for early CAG-PCI and in adjusted analyses timing of CAG-PCI was not an independent risk factor. Furthermore, a cohort study of 199 CA patients showed that exposure to iodinated contrast from CAG or CT scan was not associated with AKI. In addition, intra-arterial contrast did not increase the risk of AKI [8]. The aforementioned meta-analysis unfortunately did not report renal function [6]. Altogether, immediate CAG-PCI after CA appears to be safe and should not be postponed because of concern of contrast-induced nephrotoxicity when indicated.

## Targeted temperature management

After CA, TTM is frequently applied for neurological protection and to preserve the function of vital organs, including the kidney. Animal studies suggest that hypothermia reduces renal metabolic demand and oxygen consumption and can limit renal ischemia/reperfusion injury by suppression of oxidative stress and reduction of apoptosis. However, TTM has circulatory consequences. Hypothermia induces peripheral vasoconstriction creating a temporary relative central hypervolemia with increased renal blood flow and increased diuresis mediated by suppression of vasopressin [9]. Subsequent low circulating volume and high afterload may cause hypotension and a decrease in renal blood flow with vasoconstriction of the afferent arterioles, potentially creating a vicious circle of ongoing decrease in renal blood flow and increase of renal vascular resistance. The renal function, already impaired due to CA, can become progressively depressed.

A meta-analysis of 19 clinical studies in different populations did not find any effect of mild therapeutic hypothermia (MTH) on prevention of AKI. However, most studies were not primarily designed to investigate the effect of MTH on renal function and the definition of AKI varied substantially [10]. Only locally applied hypothermia by selective renal artery perfusion appeared to be protective for renal function in one very small (*n* = 30) RCT in patients undergoing thoracoabdominal aortic aneurysm repair compared to normothermic perfusion. Meta-regression analysis found that a lower target cooling temperature was associated with a decreased risk of AKI [11]. However, this association was predominantly due to the aforementioned study with intra-renal arterial cooling of 15 °C. [11] After removal of this study, this association did not persist.

Studies specifically addressing the effect of systemic therapeutic hypothermia on renal function in CA patients are scarce. In an explorative analysis of participants of the hypothermia after cardiac arrest trial, MTH was associated with delayed improvement in renal function, but after 4 weeks the difference with normothermic patients had disappeared [12].

Recently, a post hoc analysis of the TTM trial, which previously found no survival or neurological benefit of cooling to 33 °C compared to 36 °C [1], showed that patients in the 33 °C group needed more intravenous fluids and vasopressors, and had more AKI with worse severity compared to those maintained at 36 °C, but in adjusted analysis the difference was not significant.

In conclusion, with regard to renal function, CAG-PCI can be performed safely when indicated in CA-patients, even immediately after the return of spontaneous circulation. Mild therapeutic hypothermia is not renoprotective.

## Abbreviations

AKI: Acute kidney injury
CA: Cardiac arrest
CAG-PCI: Coronary angiography with percutaneous coronary intervention
MTH: Mild therapeutic hypothermia
STEMI: ST-elevation myocardial infarction
TTM: Targeted temperature management
